# The Springs of Conduct

**Published:** 1886-09

**Authors:** 


*The Springs of Conduct.* By C. LLOYD MORGAN, Professor of Geology and Zoology, University College, Bristol. London: KEGAN PAUL, TRENCH & CO. 1885.

The task which Professor Morgan has undertaken in this work; viz., to analyse the motives of human action and to probe the "abysmal depths of personality," is—as was said of the subclavian artery—"deep, dark, and dangerous;" and it is a proof of the careful study and knowledge he has brought to bear upon it, that it is clear, well expressed, compact, and, above all, free from that "transcendental moonshine" and "theosophico-metaphysical haze" that so often abound in books dealing in such matters. The author does not pretend to exhaust the subject; but within the limits of 300 pages, such questions as the relations of mind and body, many of the applications of the theory of evolution, the morals of conduct, free-will, determinism, feeling, and other kindred topics, are discussed with thoughtfulness and ability. The scientific training of the biologist shows itself throughout, and the explanations given or suggested are chiefly drawn from physiology and the all-pervading and generally ill-understood doctrine of evolution. At the same time, the author strongly protests against the followers of so-called materialism, "who seem to believe that the ultimate elements reached by analysis have a truer reality than the phenomena submitted to analysis." The chapters on the structure and functions of the nervous system, and the conditions of consciousness, although open in places to criticism, are well written, and show a wide familiarity with the subjects. As a metaphysician, Professor Morgan chiefly acts as an exponent of others' views, with, here and there, a refutation or objection. For example, in Chapter IV. (Part II.) he falls foul of Mr. Herbert Spencer's "Persistence of Force;" and in Chapter III. (Part III.), of the lecture given at the Bristol Museum, by the Rev. J. M. Wilson, of Clifton College, on the evolution of Ethics. In these and other instances the questions discussed will probably remain undecided in the minds of many. The ideas of Descartes, Spinoza, Comte, and others have been evidently clearly grasped, and bear fruit in these pages. It is a compliment (but nevertheless true, we think) to say that few could read the book quickly, and still fewer without an acquisition of ideas on questions which ought to interest all who lay claim to scientific knowledge.

